# Practice education facilitators perceptions and experiences of their role in the clinical learning environment for nursing students: a qualitative study

**DOI:** 10.1186/s12912-023-01328-3

**Published:** 2023-05-17

**Authors:** Cathrine Mathisen, Ida T. Bjørk, Lena G. Heyn, Turid-Iren Jacobsen, Elisabeth H. Hansen

**Affiliations:** 1 Faculty of Health and Social Sciences, Department of Nursing and Health Sciences, University of South-Eastern Norway, Post office box 235, Kongsberg, 3603 Norway; 2 Department of Nursing Science, University of Oslo, Post office box 1018, Blindern, Oslo, 0315 Norway

**Keywords:** Clinical placement, Clinical practicum, Clinical learning environment, Nursing education, Practice education facilitator

## Abstract

**Background:**

Clinical placement is recognised as essential for nursing students’ development of clinical competence. However, difficulties in providing supportive clinical learning environments are a well-known challenge in nursing education. In Norway, the use of nurse educators in joint university and clinical roles has been recommended as an initiative to strengthen the clinical learning environment and enhance the educational quality. In this study we use the term practice education facilitator in a generic sense for these roles. The aim of this study was to explore how practice education facilitators can contribute to strengthen the clinical learning environments for nursing students.

**Methods:**

This study has a qualitative explorative design with a purposive sample of practice education facilitators affiliated to three different universities located in southeast, mid-, and northern Norway. Individual in-depth interviews with 12 participants were conducted during spring 2021.

**Results:**

A thematic analysis resulted in four themes: “coherence between theory and practice”; “student support and guidance during placement”; “supporting the supervisors to support the students” and “factors influencing the practice education facilitators’ performance in their role”. The participants experienced that the practice education facilitator role contributed to strengthened clinical learning environments. However, their performance in the role was found to be contingent upon factors such as time allocated for the role, personal and professional attributes of the post holder, and a common understanding within the organisations regarding practice learning and role remits for the practice education facilitator.

**Conclusions:**

Findings indicate that the practice education facilitator role can be a valuable resource for clinical supervisors and nursing students in clinical placement. Moreover, nurse educators who are familiar with the clinical area, and who are insiders in both settings, are ideally placed to contribute to bridge the theory-practice gap. The benefits of using these roles, however, were influenced by personal attributes of the post holder, time allocated for the role and the number of practice education facilitators positions, and management anchorage. Thus, to achieve the full potential of these roles, efforts to reduce these barriers should be considered.

## Background

Opportunities to apply theoretical knowledge to real-world clinical situations is recognised as essential for nursing students’ development of the competencies needed to provide safe and high-quality patient care upon graduation [1]. Hence, clinical practice is a vital component of the nursing education curriculum in Norway, and in line with the EU directions, approximately 50% of the education consists of clinical placement [2]. Research indicates that nursing students’ opportunities to achieve their learning outcomes and their development of clinical competence are influenced by the quality of the clinical learning environment [3, 4].

A clinical learning environment has been defined as ‘any area where nursing students apply theory to practice by conducting actual or simulated patient care to gain experiential knowledge about skills, attitudes and decision-making abilities necessary to become a competent, entry-level nurse’ (3, p.508). The clinical learning environment includes four attributes that influence the quality of nursing students’ learning experiences: the physical space, psychosocial and interaction factors, the organisational culture and teaching and learning components [3, 5]. In particular, the clinical supervisor plays a key role within the clinical learning environment [4, 6, 7]. In Norway, this role is usually undertaken by staff nurses, who supervise nursing students in addition to their clinical duties [8]. They are also partly responsible for student assessment in collaboration with a nurse educator from the university. Thus, circumstances, such as high patient acuity, understaffing and a high workload, are barriers for clinical supervisors’ provision of a supportive clinical learning environment for nursing students [1, 8]. Many clinical supervisors lack formal training in their role, which may affect the quality of both the supervision and the assessment provided to students [9, 10]. Another challenge is that students’ access to relevant learning situations varies due to a high degree of specialisation in health care services [8]. These circumstances have led to concerns regarding nursing students’ development of clinical competence and their fitness for practice upon graduation [11, 12].

In Norway, the use of nurse educators in joint roles between academic institutions and placement providers has been recommended as an initiative to strengthen the clinical learning environment for nursing students [13]. A survey conducted by the Norwegian Nurses Organisation in 2021 indicated that many education providers have university lecturers in joint appointments [14]. The term ‘joint appointment’ [kombinert stilling] is used as an umbrella term for everyone who has a post in both an academic institution and a clinical area. Thus, these posts are not necessarily explicitly developed to support and strengthen the clinical learning environment. One exception is the Arctic University of Norway and the University Hospital of North Norway, which developed a joint appointment introduced in 2012. Published papers from this project have shown that these posts have been positively evaluated and perceived to be a valuable resource for clinical supervisors [15, 16].

Internationally, different joint university and clinical roles have emerged over the years to support nursing students and clinical supervisors. Examples of these roles are the lecturer-practitioner [17], the practice placement facilitator [18], the clinical placement coordinator [19] and the practice education facilitator [20]. These roles have been developed to meet individual and local needs, and thus different role remits and titles exist [21, 22].

There is a lack of more recent research literature on placement support roles. The existing literature indicates that one benefit from introducing such roles is the development of stronger links between the universities and the clinical setting [18, 20, 22–24], which in turn may contribute to enhanced quality of the clinical learning environment for nursing students [18, 19]. Additionally, these roles have been found to be appreciated and acknowledged by clinical supervisors as valuable resources, especially for those who are facing challenging student situations [16, 20]. Challenges that have been reported are lack of role clarity and lack of preparation and support for the role [25] and feelings of being overwhelmed and isolated [17, 26].

In this study, we use the term ‘practice education facilitator’ in a generic sense for placement support roles, where post holders are employed by an academic institution, a health care provider or both organisations to work within the clinical field to promote and to support clinical nursing education. A practice education facilitator provides guidance and support to both nursing students and clinical supervisors within the clinical setting and is expected to act as a liaison between the academic institution and the clinical placement site [22].

Although prior research has indicated that nursing students and clinical supervisors recognise the value of using practice education facilitators, there is a lack of empirical knowledge focusing on how such roles may contribute to strengthening the clinical learning environment in the Norwegian context.

## Methods

### Aim

The aim was to explore practice education facilitators’ experiences and views on how this role influences the clinical learning environments for nursing students.

The research questions were as follows:

1. What are the participants’ experiences of holding a practice education facilitator post?
2. What are the participants’ views on how the practice education facilitator role can contribute to strengthening the clinical learning environments for nursing students?
3. What factors facilitate or hinder the practice education facilitators’ possibilities to fulfil their role?

### Design

For this exploratory study, a qualitative approach with individual interviews was used to explore practice education facilitators’ experiences and views of holding this post. This design was suitable to gain a deeper understanding [27] of how these roles may influence the clinical learning environment for nursing students because little research has previously been conducted on these roles in Norway. The Consolidated criteria for reporting qualitative research (COREQ) checklist was applied in this study [28].

### Sample and setting

A purposive sampling approach was used to recruit participants with in-depth knowledge on the topic of interest. The purpose of this sampling approach was to select the participants that best could answer our research questions [29]. The participants were recruited through nurse education managers from three universities that used practice education facilitators in their education settings. The managers communicated the request to participate in the study from the researchers to potential participants by e-mail and were instructed to send the response to the researchers to ensure that the managers were not directly involved in the recruitment process.

A total of 28 practice education facilitators were invited to participate in the study, and 12 facilitators affiliated with all three universities agreed to participate. The participants were employed in 20–50% posts as practice education facilitators and had from one to nine years of experience (mean 4,3) in the post. The participants had worked an average of 17.3 years as nurses. Nine participants had either a master’s degree or post-degree education in nursing, and 10 participants had formal training in supervision. About half of the participants were employed both at a university and at an associated healthcare institution. The other half were employed solely by a healthcare institution, but the practice education facilitator post was affiliated with a university. All the participants worked within the clinical field in addition to the practice education facilitator post, some as regular staff nurses, while the majority comprised educational nurses.

### Data collection

Data were collected through 12 individual interviews carried out between March and June 2021. Due to hospital restrictions caused by the COVID-19 pandemic, all interviews were conducted by the first author by phone or by videoconference via Zoom or Teams. The interviews were semi-structured, and an interview guide developed by the authors structured the interviews to cover four areas: (1) Role remits, (2) Value of using practice education facilitator roles in clinical nursing education, (3) Factors influencing the value and (4) Personal experiences of holding the role. Follow-up questions were asked to obtain in-depth information from the participants. The interviews lasted from 45 to 75 min and were audio-recorded. According to Malterud [30], information power in qualitative research is dependent on design and aim, and that a larger sample size is needed for studies with broader aims. This study, however, had a specific aim with a homogenous sample and the collected data was rich. Thus, the authors agreed that 12 informants were sufficient in this study.

### Data analysis

A thematic analysis was used, following the six recursive phases described by Braun and Clarke [31]: familiarising; coding; generating initial themes; reviewing and developing themes; refining, defining and naming themes; and writing the report. The first author transcribed the interviews verbatim shortly after they were conducted, and the software programme NVivo (version 1.6) was used to manage and structure the text. The analysis process was inductive. To become familiar with the data, the authors read and reread the transcripts several times and noted initial ideas. A selection of the transcripts was then coded by three of the authors (CM, ITB, EHH), and the codes were compared to calibrate the process. To preserve the context, the surrounding data for each code were included. The codes were further organised into initial themes by the first author before all authors reviewed the themes by checking the transcripts within each theme to determine whether the data corresponded with the potential themes. This resulted in a process of moving back and forth within the entire dataset, as described by Braun and Clarke [31]. During this phase, we generated some new codes, and some codes were reorganised into new potential themes. Finally, each theme was refined by generating clear definitions and suitable names. An example of the data analysis process is presented in Table 1.

**Table 1:** Example of the thematic analysis process

Meaning units	Code	Subtheme	Theme
I believe that it’s important as a practice education facilitator to find a place in the space between the university and the clinical site to bridge the gap between theory and practice, I have been very interested in this issue for many years [P6]	Acts as a liaison between education and placement	Enhancing the collaboration between the university and the placement provider	Coherence between theory and practice
At the university, I can tell them what is possible to do in placement and not. In a sense I’m the realistic one. I believe that it is very important that both sides are represented in these meetings. [P7]			
By being present at campus and becoming familiar with what they do there, I can connect learning situations in placement to learning activities the student had at campus prior to the placement period. [P 12]	Connects learning activities at campus to learning situations in placement	Facilitates coherence between learning activities at campus and learning situations in placement	
I talk with the supervisors about the physical assessment skills that the students learn at campus and how we can provide relevant learning situations for them to practice on these skills in placement. [P2]			

### Ethical consideration

The Norwegian Centre for Research Data (NSD) approved the study, project number 255,238. The participants received both written and oral information about the study, and informed written consent was collected from all participants. Furthermore, the participants were informed that they could withdraw from participation at any time, without this leading to negative consequences. Confidentiality was secured by not mentioning the participants names, workplaces, or other identifying information in the transcripts.

## Results

The key findings generated from the thematic analysis resulted in four themes: ‘coherence between theory and practice’, ‘student support and guidance during placement’, ‘supporting the supervisors to support the students’ and ‘factors influencing the practice education facilitators’ performance in their role’. An overview of the themes and subthemes are presented in Table 2. The following descriptions of the themes include quotations from the interviews to share the participants’ voices.

**Table 2:** Overview of subthemes and themes

Themes	Subthemes
Coherence between theory and practice	Enhancing the collaboration between the university and the placement provider
	Facilitates coherence between learning activities at campus and learning situations in placement
Student support and guidance during placement	Supporting students who are facing challenges during placement
	Influencing the culture for practice learning
Supporting the supervisors to support the students	Clinical supervisors become more secure in their role
	Improving student assessment
	Students who are facing challenges in placement are more likely identified
Factors influencing the practice education facilitators’ performance in their role	Flexibility in the clinical role influences the performance in the practice education facilitator role
	Familiarity and position in the clinical setting influence practice education facilitators’ ability to effect changes

### Coherence between theory and practice

The participants expressed that a key role remit as a practice education facilitator was being a liaison between the university and the placement site. They collaborated closely with educators from the university, clinical staff, and managers in placement. Their familiarity with both settings enabled them to illuminate perspectives from ‘the other side’, which contributed to an enhanced understanding and collaboration between the organisations.

I can communicate and argue in a different way because I have first-hand experience from both places. [P11]

By being insiders in both settings, the participants were also able to identify inadequate coherence between what the students were taught in the academic setting and the clinical reality that the students faced during their placement. One example that was mentioned by several of the participants was a mismatch between the physical assessment skills curriculum at the university and how physical assessments were performed in the clinical setting:

The students become very insecure when they experience that no one is doing the physical assessment the way they have learnt in the clinical setting. [P1]

To strengthen the coherence between what students were taught in the two settings, it was important that the clinical supervisors were familiar with the student’s syllabus for the placement period. Furthermore, it was crucial that the syllabus was in accordance with the clinical reality and viewed as relevant by the clinicians. Thus, an important task for the participants was to facilitate a common understanding between the academic setting and the clinical setting.

I have had the role for about a year now and my views about the nursing education have changed. I didn’t see the whole picture as a clinician. So, an important task for me in the role is to improve clinicians understanding of what happens at campus as well as help nurse educators understand the reality in practice. [P10]

Some of the participants used their first-hand knowledge from the clinical field to enhance the clinical relevance of the didactic sessions at the university, which in turn could contribute to students being better prepared for placement. Some also expressed that the practice education facilitator post had improved their understanding of the nursing education, which was helpful when they prepared clinical supervisors for their role, such as by helping clinical supervisors understand and connect the learning outcomes described in the syllabus to clinical learning situations during placement.

### Support and guidance provided to students during placement

Some participants explained that their role was crucial when organisational challenges occurred during clinical placement. For instance, if a student needed a new placement site, they organised the transfer and ensured that the student was given the placement that was required. In other cases, when students lacked access to relevant learning situations, they organised alternative learning activities for these students. They also provided guidance and supervision to individual students who faced challenges during placement, such as personal problems or relational challenges with the supervisor:

There are students that have told me that they felt unwanted at the ward and that they would not have made it if they had not received the support from me. [P2]

The participants had an important task in being champions for practice learning within the clinical area. If someone expressed negative feelings regarding nursing students during placement, they focused on reminding nursing staff of the importance of doing a good job with the students because it is crucial for recruiting new nurses. Some participants also tried to influence the culture for practice learning in the wards by increasing managers’ understanding of nursing students needs and by developing a common understanding of the resources needed for student supervision:

I have worked with the managers to help them realise that supervising students is something that takes time, and this has resulted in allocated time for supervision. [P11]

Because patient care is the focus for managers within the clinical area, there was a need for someone who helped them keep in mind that research and education are also tasks they are obliged to accommodate. Some participants expressed that they contributed to influencing the awareness of practice learning and clinical placement amongst higher-level managers because they had a seat in key management meetings.

### Supporting the supervisors to support the students

The participants stated that the clinical supervisors appreciated the guidance and support they provided. The group sessions that most of the participants offered provided a space where clinical supervisors had opportunities to focus on their supervisor role, which is valuable in a setting with a heavy clinical workload and a lack of dedicated time for student supervision. Topics such as ‘how to succeed as a supervisor’, ‘what kind of supervisor are you’ and ‘what challenges do you face in the supervisor role’ where often brought up. The group sessions also provided opportunities for the participants to inform and update the clinical supervisors on more practical aspects of the role, such as the syllabus, assessments, and regulations.

I believe that it is very important to offer support and guidance which is easy to access. The nurse educators are very busy and without access to a practice educator facilitator the clinical supervisors must solve many things on their own. [P7]

The participants experienced that it was important to be present in the clinical area and to use opportunities that arose to give constructive feedback to clinical supervisors in relation to actual student supervision. One participant provided an example regarding the quality of the supervision provided to students, which had improved after the implementation of the practice education facilitator role:

Many of the supervisors are used to just telling and showing the students how to do things. Now, they try to support the students to figure it out themselves instead. I believe many have changed a bit—changed in the way they supervise students. [P10]

The participants who offered group supervision to clinical supervisors during placement periods stated that these meetings facilitated networking between clinical supervisors, which in turn extended their support system. Networking with other supervisors and opportunities to share experiences and seek support and guidance in the group supervision meetings helped clinical supervisors feel more secure in the role because they had support:

The others provide new eyes on the situation, and you get confirmation: ‘Yes, I agree with you in that assessment’. You get the opportunity to calibrate in a sense. [P4]

During group sessions, supervisors often expressed that they were unsure of what they should expect from the students regarding practical skills and theoretical knowledge. Furthermore, they needed guidance on how to use the assessment tool to assess student performance in placement and to understand the learning outcome descriptions provided by the university.

The participants were regularly involved in situations where clinical supervisors found that their students struggled to achieve the learning outcomes. In these situations, some participants preferred to spend some time with the students themselves at first to perform an independent assessment of the student. It is important that they provided a second opinion in these situations, especially when students were at risk of failing the course. It is also important to provide support to the supervisors because failing a student is considered a huge responsibility. Lack of timely support and guidance in these situations could lead to a ‘failing to fail’ situation because a ‘second eye’ was not provided before midterm assessment. Some participants also mentioned that the clinical supervisors need ongoing support and guidance to manage assessments in a proper way and that they have an important task in contributing to the enhanced quality of the student assessment process:

I have the impression that when the workload is very high, the students are given tasks they need to do independently, and later, their assessment tends to be based on how efficiently they solve the tasks that are given to them instead of asking them questions and evaluating how they reflect. [P3]

In addition to the provision of guidance and support, group supervision meetings were also important arenas for promoting the practice education facilitator role and building relationships with clinical supervisors. The participants explained that it is easier for clinical supervisors to seek support and guidance from someone they know and someone who is easy to access compared to the academic nurse teachers, who are not geographically located at the same place. Furthermore, they noted that ad hoc supervision is important for clinical supervisors who experience challenging situations, such as students at risk of failing. Not feeling alone in these situations and having access to support and guidance helped the supervisors trust their own judgments, and it became easier for them to act when they faced challenges.

Some participants commented that it could be difficult for clinical supervisors to admit that they were facing challenges because this could reflect on their shortcomings as a supervisor; however, the trusting relationship they developed with each other could help them be willing to admit that they are facing challenges:

She received a lot of support from the group, and then, after a while, she shared more and more in a way. Because basically, they often feel that they are the ones to blame in a sense. [P10]

Some of the participants stated that they believed the introduction of the practice education facilitator role had contributed to earlier identification of students facing challenges. This, in turn helped to increase the possibilities for being able to do something about the situation before it is too late for the student, decreasing the risks of ‘failing to fail’ situations.

### Factors influencing practice education facilitators’ performance in their role

Being accessible and visible within the clinical area was stated by all participants as essential to performing the job. Their clinical post could cause challenges for the participants, who stated that they had little influence over their own workplans. Those with opportunities to adjust their workplans viewed this flexibility as an advantage. Thus, having a manager that understands their need for flexibility regarding their workplan is crucial:

If I have an important meeting, let me attend that meeting without feeling guilty because I am leaving the ward. They need to understand that I need that flexibility to do my job. [P11]

Several participants experienced that ward managers who did not recognise the importance of strengthening student supervision or the benefits of the practice education facilitator role did not prioritise sending their staff to group supervision meetings. When there was a shared understanding and managers ‘were onboard’, they said that the clinical supervisors were more likely to attend the group meetings. Thus, a common understanding and anchorage amongst managers at all levels are crucial. Furthermore, the participants expressed that their position within both education and the placement site is important:

If I was a regular staff nurse, then I believe that this job as a practice education facilitator would have been much more difficult because I wouldn’t have had that voice within the organisation. [P6]

Most participants were experienced nurses familiar with systems and key persons at the placement site. This provided credibility and acceptance as insiders within the clinical area, which is essential to developing trusting relationships with staff and managers. Having a position within the clinical setting provides opportunities to communicate with managers and to be listened to in a different way than without this position. Some explained that their position helped them have access to the clinical environment, which is necessary to be able to provide ad hoc supervision within the clinical area. Familiarity with the different wards and personal relations broke down the communication barrier with staff and managers. Others, who did not have the same network or many years of experience in the placement site, stated that it was crucial for them to spend time within the field to develop relationships with clinical staff and managers to be accepted as an insider.

## Discussion

The current study is the first to report findings on practice education facilitators experiences and perceptions on how this role can contribute to strengthened clinical learning environments for nursing students in the Norwegian context. Our findings indicate that practice education facilitators can contribute to strengthening the clinical learning environment, but the benefits of using these roles are contingent upon certain factors, such as time allocated for the role, the personal and professional attributes of the post holder and a joint agreement and management anchorage at all levels.

All participants in this study pointed out that being a liaison between the university and placement was a key role remit for a practice education facilitator. This is in line with international studies on similar roles that were conducted with a specific aim of bridging the theory–practice gap [32]. Nursing students spend their time between the academic setting and clinical placement and having two segregated units in learning leads to challenges in creating coherence between the two. Thus, experiences of a gap between what students learn at the university and what happens in placement is a well-known challenge in nursing education that is often referred to as the theory–practice gap [33, 34].

In a concept analysis, the attributes of the theory-practice gap were found to be relational challenges between the university and clinical placement, practice failing to reflect theory and theory being perceived as irrelevant to practice [33]. Thus, to synchronise what is taught at the university and what students are offered in placement, there is a need for an effective collaboration between the two settings [34]. In this study, we found that the participants were ideally placed to contribute to enhanced collaboration between the placement provider and the university and to contribute to enhanced coherence between theory and practice; however, to what degree the post holder was enabled to effect changes is not known and was likely influenced by the factors found to influence the practice education facilitators’ possibilities to fulfil their job. To build relationships and develop effective collaboration, findings from previous research have shown that it is essential that there is a common agreement and that both settings acknowledge the benefits of developing a good collaboration [34].

The findings from this study indicate that practice education facilitators can contribute to a strengthened quality in clinical placement by influencing several of the components described by Flott and Linden [3] as attributes of the clinical learning environment. Particularly the attribute ‘teaching and learning components’, which involves the effectiveness of clinical supervision and students’ access to relevant learning situations. The effectiveness of clinical supervision was enhanced by practice education facilitators’ both by supporting students and by supporting clinical supervisors to support students. The participants were also found to influence the attributes ‘psychosocial and interaction factors’ and ‘organisational culture’. For instance, by being champions of practice learning, the participants influenced clinical supervisors’ attitudes toward nursing students, which can be linked to the attribute ‘psychosocial and interaction factors. Flott and Linden [3] found in their concept analysis that the ‘organisational culture’ is often influenced by managers’ views on nursing education, which in turn influences clinical staffs’ behaviour regarding practice learning. This is in line with findings from this study, although some participants expressed that they were able to influence managers views and attitudes regarding student learning in a way that had helped them in fulfilling their role.

A factor that was found to influence the practice education facilitators’ performance in their role in this study was the post holders’ time allocated to the job and their opportunities to be visible and accessible in the clinical learning environment. We found that the participants who worked as regular staff nurses were more likely to experience competing demands due to having joint roles compared to those who had an educational nurse post and had more flexibility in their clinical role. Similar findings have been reported in prior research, where competing demands have been reported to influence practice education facilitators’ responsiveness to challenging situations in the clinical learning environment [37]. Practice education facilitators’ visibility and accessibility within the clinical learning environment have also been found to influence whether the role is perceived to be valuable by clinical staff and students [20, 23]. Thus, competing demands and workloads that hinder practice education facilitators from being visible and accessible in the clinical area are potential barriers to the achievement of the intended outcomes of such roles.

In this study, we found that practice education facilitators’ performance in their role was influenced by the post holders’ position and familiarity within both the clinical environment as well as the academic setting. Knowing the right persons and being someone that people listen to is necessary to effect the intended changes. Similar findings have been reported in prior research, where familiarity with both the clinical setting and the educational programme is vital [28, 35]. Personal attributes, such as good communication and interpersonal skills, have also been linked to the success of the role in prior research [36].

The existence of a common understanding regarding the practice education facilitator’s role remits and to what degree the role is anchored amongst managers was found to be a key factor that influenced the benefits of the role. For instance, managers’ familiarity with the role and their understanding regarding potential challenges when having a joint position were found to influence the participants’ ability to be visible and accessible within the clinical area. Ward managers’ familiarity with the role in addition to their attitudes towards practice learning were also found to be potential barriers to initiatives carried out by practice education facilitators. Thus, to achieve the intended outcomes of the role, it is crucial that there is a joint agreement at all levels and that managers are involved and have ownership of the initiatives implemented within the organisation. Challenges in achieving real-world changes in hospitals are well known [37]. Hospitals are complex organisations with a unique organisational culture, and there are multifaceted barriers to success when initiatives, such as the role of the practice education facilitator, are implemented [38, 39]. Patient care is a prioritised task, and hospitals are known to have a high clinical workload [40], which also can explain difficulties in prioritising practice learning in some areas.

### Limitations and strengths

Although this study provides valuable insights into how the practice education facilitator role may influence the clinical learning environment for nursing students, a limitation of this study is that the participants evaluated their own role, which may potentially bias the experiences they choose to share with the researchers. However, the participants were informed during the interviews that there were no right or wrong answers, and the researchers felt that the participants were nuanced when they shared their experiences. While we did explore practice education facilitators’ experiences and views, we also acknowledge that this study was carried out in Norway and may not reflect the practice education facilitator role in other countries; however, our findings are in line with prior research on similar roles conducted in other countries. A strength of this study is that we included participants affiliated with different universities that also were geographically spread out within the country. The research group who carried out this study was assembled to ensure methodological competence in conducting a qualitative study, knowledge of theoretical and practical aspects of nursing education and opportunities and challenges in facilitating an effective clinical learning environment for nursing students.

## Conclusions

This study has highlighted how the practice education facilitator role can contribute to strengthened clinical learning environment for nursing students, although the participants in this study experienced several barriers that influenced their performance in the role. In particular, they experienced that competing demands,, and managers’ familiarity and understanding of the role influenced their opportunities to perform well.

Although the participants discussed several barriers that influenced their performance negatively, our findings demonstrate that the practice education facilitator is ideally placed to contribute to bridging the theory–practice gap. However, to achieve the full potential of these roles, certain factors, such as the personal attributes of the post holder, time allocated for the role and/or the number of practice education facilitator positions and management anchorage should be considered.

### Implications for practice and research

The practice education facilitator role is vulnerable to role strain due to a broad area of responsibilities and affiliation to two different organisations. Thus, to prevent burnout, it is necessary to ensure that these roles are appropriately supported. Moreover, it is necessary to consider the employment and management of these roles. The academic institution and the health care organisation should work together to establish clear expectations and guidelines for their responsibilities.

There is a need for further evaluation of the practice education facilitator role and future research should explore the impact these roles have on nursing students’ clinical learning environment.

## Data Availability

The data from the audio-recorded interviews used to support the findings of this study are available from the corresponding author upon reasonable request.
